# Role of Leadership in Adoption of Blockchain Technology in Small and Medium Enterprises in Saudi Arabia

**DOI:** 10.3389/fpsyg.2022.911432

**Published:** 2022-05-04

**Authors:** Nasser Alshareef, Muhammad Nawaz Tunio

**Affiliations:** ^1^College of Business Administration (CBA), Majmaah University, Al Majma’ah, Saudi Arabia; ^2^Department of Management Science, Mohammad Ali Jinnah University, Karachi, Pakistan

**Keywords:** SME’s, financing, blockchain, Saudi Arabia, distributed ledger technology (DLT)

## Abstract

This research aims to determine the role of the leadership and potential benefits that blockchain adoption may bring to SME financing in the Kingdom, as well as the foreseeable challenges that may hinder small businesses from benefiting from the adoption of blockchain. It is interesting aspect to see how leadership manages to adopt new changes amid several challenges and threats. This article also outlines policy and regulatory trends that SMEs can save operating costs and improve efficiency, thereby increasing transparency and easier access to funds. Digital technology and creative business models have the potential to help narrow the financing gap for SMEs. E-commerce and the sharing economy provide SMEs with more market access and new business models, as well as the data-driven business prospects generated through data sharing under the framework of open banking. This study provides recommendation that there is a dire need to pay attention on the complete mechanism of the SME’s in order to support them as well as promote them to show their distinction in the contribution of social and economic development. This study provides implications for the financial institutions, government agencies and society to come forward equally for the common interest.

## Introduction

Leadership has important role in adopting new changes in the organization and deal with challenges, but it is becoming more interesting to find the role of leadership in the SMEs in different functions and innovation. Across several dynamics, finance has been burning issue at SME’s level (Kautsar et al., 2018). Thus, financing has always been a thorny issue in general as well as in case of the Small and Medium Enterprises (SME’s; Sawy and Bögenhold, 2022). In some countries, 50% of SME financing applications are rejected. Some studies have found that one of the main reasons for the failure of the traditional financial industry is the slow and rigid loan process (Saito and Tsuruta, 2018; Barkley and Schweitzer, 2020).

Saudi Arabia Government leadership has adopted new emerging technologies at individual as well collective level in which greater interest is paid in the adoption of the blockchain technology. Across different sectors, actors, institutions and individuals are directed to integrated blockchain technology in the financial and government service sectors (Alilyyani et al., 2022). However, different strategies are set for the development in areas such as finance, government strategy, project planning, and lawmaking that suggest that blockchain will play an influential role in the development and modernization of the Saudi’s government, commercial, and financial sectors in the future (Syed et al., 2019).

In order to finance Saudi SMEs, Saudi policy makers should pay attention to the institutional determinants that hinder investors from financing Saudi SMEs through new methods such as blockchain, especially investors in developed countries. One of the main goals of Saudi Vision 2030 is to decrease future unemployment, the goal is to attract more investment, provide value to the economy, transfer technology, and hire more Saudis (Mason, 2021).

The International Finance Corporation estimates that among the 40,000 small and medium-sized enterprises around half of the firms in the world have unmet loan demands of between 2.1 trillion and 2.6 trillion dollars. For entrepreneurs and business owners wanting to fund development, working capital, or seasonal inventory increases, these numbers are meaningless (Coulibaly, 2019).

This problem is serious in developing countries, but it is also frustrating that developed economies lack credit channels and have comprehensive financial transactions facilities. Due to the fact that small and medium-sized businesses employ the majority of the world’s workforce, a lack of access to finance will result in slower overall growth and fewer employment (Andersson, 2021). SMEs must rely on equity and other sources of funding even in a market with exceptionally low and negative interest rates. In Switzerland, there is a significant credit gap, with 68% of SMEs unable to access any type of bank loan. Micro, small, and medium businesses are the major drivers of economic activity (SMEs). Though, whether in developed or developing economies, the availability of credit for these companies is very limited.

Current SME lending options were examined in this study. In particular, how blockchain and distributed ledger technology (DLT) may improve capital availability and close credit gaps. Innovations in financial technology and finance methods are required to enable more SMEs to get loans. Lenders will be able to give credit to borrowers that are now deemed poor thanks to blockchain-based apps that track payments, contract fulfillment, and many other elements of company activity. Standardized financial instruments produced natively on the blockchain will enable hitherto unachievable near-real-time reporting and securitization of loan books.

Blockchain is no longer just Bitcoin. With the rapid development of enterprise-level solutions, we have witnessed a proliferation of blockchain use cases in the banking industry in the Kingdom of Saudi Arabia (Reinsberg, 2021).

Financing for Saudi SMEs, it discusses blockchain technology and its implications for the financial industry. After the global financial crisis, Bitcoin developed as a blockchain technology. Bills were used to authenticate transactions through the mining process at the time, and blockchain technology was a peer-to-peer, totally decentralized or distributed network of bills (Khan and Byun, 2021).

Developers may now simply construct decentralized apps on top of the current network, thanks to the highly programmable Ethereum. Simultaneously, the notion of token economics has grown in popularity. Rather of utilizing traditional economic incentives, encrypted payouts are utilized to encourage stakeholders in the blockchain ecosystem, according to this idea. Bitcoin and Ethereum are both public and open blockchain networks that are appealing to retail users, but they are still a long way from being conventional financial instruments. As a result, many Wall Street veterans and technological titans have recognized the financial potential of blockchain. As a result, they’ve developed several new blockchains that are more in accordance with rules and regulations, are more private, and are more suited to corporate applications. Hyperledger by IBM and Corda by R3 are two good examples. Small and medium-sized businesses spend 28 times more in blockchain than large businesses, according to a research performed by Emory University (Atlanta, GA, United States) in conjunction with Provide Technologies and Aprio. The majority of blockchain-based initiatives attempt to automate corporate operations, according to the report, and identity verification and compliance are the world’s second and third largest blockchain uses, respectively (Kunhibava et al., 2021).

According to the findings, the payment industry ranks sixth in terms of blockchain adoption, with identity management and market governance following closely behind. In the Netherlands, for example, SMEs account for more than 90% of all businesses, and they account for 60% of the economy’s added value (Marques, 2021). More and more blockchain solutions are being developed by creative start-ups focusing on SMEs. But, in addition to financial technology, the areas where DLT really makes sense may be those where existing SME’s lack sufficient understanding of how DLT works and how they adopt these technologies (traditional small and medium-sized enterprises).

Blockchain (corrupt and decentralized ledge) will create a new and simple way to finance small and medium enterprises in Saudi Arabia. Recent research predicts that technological advancements such as blockchain will create new opportunities for global finance for SMEs in the next coming years. Blockchain is a financing revolution because it is a faster, safer, and more effective way to finance small and medium-sized enterprises in Saudi Arabia.

### Objectives for Proposed Research

The study’s goal is to figure out what institutional barriers are preventing investors from using Blockchain to fund Saudi SMEs, as well as how important Blockchain is to them. The goals of this study are to:

i. Establish the institutional factors that prevent investors from financing Saudi SMEs using blockchain; and to discover the institutional factors that prevent investors from financing Saudi SMEs via Blockchain.
ii. To examine how Saudi SMEs would perceive and adapt to the Blockchain and other challenges in the KSA environment.

To assess the Kingdom of Saudi Arabia’s potential to use Blockchain to fund SMEs and their institutional policies.

## Theory and the Review of Literature

**FIGURE 1:**
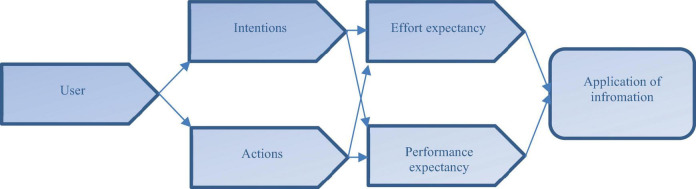
UTAUT model.

A very famous and interesting (Figure 1) unified theory of acceptance and use of technology (UTAUT) was introduced by Venkatesh et al. (2003) and this theory provides ground for the study with respect to the blockchain technology, constraints in the process and lack of support from the concerned sources. However, this theory is based on the different eight models in the domain of the information technology in which different components are integrated from different theories like the Innovation Diffusion Theory (Moore and Benbasat, 1991; Rogers, 1995); the Technology Acceptance Model (Davis, 1989); the Combined Technology Acceptance Model and Theory of Planned Behavior (Taylor and Todd, 1995); the Model of PC Utilization (Thompson et al., 1991); the Theory of Planned Behavior (Ajzen, 1991); Theory of Reasoned Action (Fishbein and Ajzen, 1975); the Social Cognitive Theory (Bandura, 1986); and the Motivational Model (Davis et al., 1992).

The UTAUT entails the intentions and interest of the user to use information technology and information system because the UTAUT is claimed that it clues about the effort expectancy and performance expectancy as a sign of predictors of intentions and actions toward application of information in different forms and for different reasons (Adeola and Evans, 2017; Evans, 2019). Several studies have acknowledged and supported the theory (e.g., Williams et al., 2015; Celik, 2016; Bala Subrahmanya, 2017; El-Masri and Tarhini, 2017; Howard et al., 2017).

The basic factors in business are transactions and trading which rely on the trust and goodwill between two or more individuals or enterprises (Kalogiannidis and Chatzitheodoridis, 2021). Thus, trust plays pivotal role in the all kinds of financial instruments and strategies to initiate, maintain and grow in business. Financial system contains a trust rating system which determines that where user can be trusted. Thus, it rates user depending on her or his borrowing and history of repayment, status of credit, approved or disapproved loan, or credit limit (Sharma et al., 2019).

Furthermore, situational theory paves way for the implementation of UTAUT Model as there is no single right approach to lead because the external as well as internal dimensions of the surrounding and environment need the leader to manage to particular situation to adopt blockchain technology and implement it at grassroot level (Nawaz and Khan, 2016; Bögenhold, 2018). Thus, adoption of the emerging technologies like blockchain is a need of hour where strong leadership is required to lead at organizational, and individual level (Hisrich et al., 2007; Sharif and Ghodoosi, 2022).

The combination and merger of finance and technology is somehow marriage in between them, thus called as FinTech, these contain combined reactions and create a multiplier effect (Audretsch et al., 2016). FinTech ejects two streams, in the first, it is pace of exchange driven by Big Data, machine learning, commoditization of technology and Artificial Intelligence (AI). However, second stream indicates that new non-financial firms have embarked financial service business and invested in it (Fellenz et al., 2009).

However, FinTech entails another two dimensions as well in which it shows about the traditional SMEs transform by adopting emerging technologies (Pizzi et al., 2021). Traditional enterprises such as Ping An Group, Industrial and Commercial Bank of China, Morgan Stanley and Goldman Sachs, use Big Data and other new technologies to upgrade and transform their service. Thus, the second dimension is that technology enterprises use emerging technologies to develop financial services, such as the initial motive of Facebook, Apple, Google, Ant Financial (China), Jingdong Finance (China), Tencent (China) was not to deal financial transaction or earning money but later their discovered and developed their own platforms of financial services to satisfy customers’ needs and create new forms of entrepreneurial finance landscape (Chen, 2016). Micro, small and medium-sized enterprises (SMEs) are the integrated part of the strategic planning to attend economic growth in the Middle East, North Africa, Afghanistan and Pakistan (MENA) region. Across these regions, countries vary in different ways, like young people have mostly unemployed, government sector has limited capacity to accommodate youth and provide employment to them, unstable price mechanism, and income disparities. In order to deal with such situations, different actors and institutions try to catalyze the growth of SMEs and startups in order to create the new jobs. In this regard, digital technologies are a game changer in promoting and boosting of the SMEs (Bracci et al., 2021).

Bracci et al. (2021) conducted a exploratory study in which they analyzed the factors that transform to digitalization and lead SMEs to adopt blockchain. They analyzed the implementation of blockchain technology and found various factors like role, age, gender, and perceived usefulness and ease of use of blockchain.

Blockchain knowledge is relevant for increased interest in its diffusion and use. However, more work is needed to enhance the level of knowledge and to increase the level of perceived usefulness and ease of use for SMEs (Tobias, 1994). This must be done considering the problem of knowledge hiding between collaborators and employees. In this sense, the emotional and cognitive aspects are important to enhance and diffuse blockchain knowledge (Chang et al., 2020). The evolution of the blockchain and its knowledge used for the business purpose has been supportive for the SMEs because the blockchain assists and accelerates knowledge management in the different departments and whole organization (Chaithanapat and Rakthin, 2021).

## Research Design

This is a desk research project in which the literature on the issue is evaluated in order to arrive at the best conclusion possible. Furthermore, content analysis is used in this study because it is flexible in its nature to analyze the text data and to achieve the objective of the study (Downe-Wamboldt, 1992). Content analysis approach is very helpful in illuminating key issues and interpreting the content and context of the text (Ferrell et al., 2000). Mainly, this qualitative approach is helpful in providing knowledge and understanding the phenomenon being studies (Kondracki and Wellman, 2002). In this regard, data for this study was largely gathered from secondary sources, which included research publications, SME industry reports, books, a variety of websites, trade journals, newspapers, and magazines. is a type of desk research in which the literature on the issue is studied in order to arrive at the best conclusion. However, data for this study was largely gathered from secondary sources, which included research publications, SME industry reports, books, a variety of websites, trade journals, newspapers, and magazines.

## Discussion

In order to finance SMEs in the region of the Saudi, institutions responsible for designing policies and programs should pay attention to the institutional determinants that hinder investors from financing Saudi SMEs through new methods such as blockchain, especially investors from developed countries. Blockchain will create a new and simple way to finance KSA’s SMEs. Recent research predicts that technological advancements such as blockchain will create new opportunities for global finance for SME’s in the next coming decades. Blockchain is a financing revolution because it is a faster, safer, and more effective way to finance SME’s within the Kingdom of Saudi Arabia.

With current emphasis and attention on diversification to reduce dependence on future oil revenues, generally speaking, the private sector, especially small and medium-sized enterprises, may be regarded as a key pillar of a diversification strategy and leading the economy.

Obtaining cash is a frequent issue for small and medium businesses (SMEs), particularly Saudi Arabian SMEs operating in the country. Domestic banks are more likely to pick state-owned companies as a safe bet. SMEs are the backbone of the economy in many developed as well developing countries. SMEs are believed to reduce unemployment by creating new employment opportunities in different capacities of all age groups. Thus, these small businesses are critical to global economic and social growth. Most SMEs are service-oriented and family-owned, and they spend little in capital goods, reflecting the financial/accounting industry’s deficit in tangible company assets. This is due to the high risk and low creditworthiness associated with existing bank and financial institution lending criteria.

As a result, SMEs are less likely than large corporations to get bank loans, relying instead on internal finances or cash from friends and family to establish and run their enterprises. Furthermore, SMEs face industry-specific problems such as a lack of information needed to successfully conduct business and grow operations, or inefficient procedures necessary to process payments and hire other auxiliary services internationally.

It is important to acknowledge that there is little research on the institutional factors hindering SME financing through blockchain in Saudi Arabia and the Middle East and North Africa. Therefore, it is envisaged that this study will contribute to closing a significant gap. The available research on the MENA area is relatively sparse, and empirical support for the issues stated is lacking. This gives existing research rationale and basic concepts, as well as emphasizing its contribution to the literature. The main contribution of this research is to clearly understand the impact of institutions on financing policies through blockchain. In this case, limited empirical work has been done through the financing policy of blockchain cooperation with institutions and its impact on the growth of SMEs in the MENA region.

This research can also provide some other important contributions: First, its contribution to understanding blockchain technology and for sure it will also support KSA to realize its 2030 Saudi vision, whose main goal is to attract more investment to help achieve economic diversification and get rid of its dependence on oil. Second, the owners of SMEs can use the results of this research to understand and understand the environment in Saudi Arabia. This comprises SMEs that are already doing business in Saudi Arabia and/or those that may want to invest in the future.

Final recommendations of this research can be regarded as the reference basis for the conceptual development and institutional reform of government agencies (such as the General Administration of SME’s and the Minister of Commerce and Investment).

### Determinants of Kingdom of Saudi Arabia Institutions (Banks) Using Blockchain Technology

Ripple blockchain technology was utilized by Al Rajhi Bank, the world’s largest Islamic bank, to conduct safe cross-border transfers. This is the first time the technology has been successfully deployed in Saudi Arabia. A remittance between Riyadh and the Jordanian headquarters of Al Rajhi Bank was the most recent transaction. This achievement emphasizes Al Rajhi’s banking network’s technological integration and confirms the bank’s changed customer experience and continuous efforts to digitize the client’s banking experience. In Saudi Arabia, the blockchain project is part of the digital transformation of Al Rajhi Bank. The solution will provide a first-class customer experience by shortening delivery time, lowering costs, and improving accuracy.

Al Rajhi Bank continues to develop and provide industry-leading banking services, and this achievement validates the bank’s current operations. This solution lowers the cost of bank settlement and reconciliation while also increasing the predictability and efficiency of same-day liquidity management.

The Kingdom of Saudi Arabia is the world’s second-largest recipient of remittances. With more than 200 remittance centers around the kingdom, Al Rajhi Bank is the region’s biggest remittance bank. To make this technology broadly available to its retail and business clients, the bank is collaborating with the Saudi Arabian Monetary Agency (SAMA).

### Encouraging Small and Medium Enterprise Financial Growth: China-Canada Analysis

There is no need for token economics to reward behaviors since modern technologies employ alternative mathematical computations and cryptography in financial transactions. The greater issue, on the other hand, is scalability, since more isolated networks emerge, more bridges for interoperability must be built, and as more players join the network, further routes for private communication must be developed. SME development has now progressed to the third level. At this stage, it has been noted the growth of specially constructed blockchains, which are technologically agnostic and specific in the entire kingdom.

The impact of blockchain technology on the banking and financial technology industries. Nanopay is a Canadian software payment firm based in Toronto. Nanopay won the annual blockchain award from the Canadian financial technology community just a year ago. Nanopay’s system is based on a controlled blockchain ledger, which eliminates the need for consensus. As a result, their blockchain technology is extremely scalable. Nanopay’s technology also speeds up processing 30 times faster than traditional DLT blockchain solutions. Nanopay also works with digital fiat cash, which eliminates the need to convert to encrypted currency at any point throughout the transaction. Furthermore, there are certain misconceptions concerning blockchain technology that people should be aware of in order to properly comprehend their technology. People should be aware of the many changes in blockchain technology, such as whether it is decentralized or centralized, whether it is unlicensed or licensed, and if it is a public or private network. Blockchain’s appeal is that it is immutable, transparent, synchronized, cost-effective, and offers a wide range of applications. In truth, the blockchain has issues with scalability (centralized blockchains are not an issue), interoperability (cross-chain technology firms assist with integration), and legal framework (regulatory policies need to keep up with new technologies). With these concerns in mind, Nanopay has created the Liquid product line, which is based on a controlled blockchain. Cash management and reconciliation, liquidity management solutions for SMEs, and guaranteeing faster and cheaper cross-border payments are all use cases for the product.

The final key lesson is that blockchain is an innovative tool that is extremely helpful and powerful when used alone, but much more so when combined with other technologies such as AI, machine learning, APIs, and virtual asset management.

Dengfeng Zhang of Linklogis, a Chinese financial technology firm, shared his thoughts on blockchain-based supplier finance solutions. In a normal supply chain, when suppliers’ size shrinks, it becomes increasingly difficult for them to secure finance from the system. Because of their vast scale, access to information, and lack of liquidity, banks are most eager to collaborate with Tier 1 suppliers. However, N-tier providers (lower-tier suppliers in the chain) have relatively few collaterals, and tracing the interaction with anchor customers is difficult. As a result, N-tier suppliers have a tough time obtaining funding from banks. Linklogis’ supply chain finance solutions, such as Deep-Tier Financing, are based on blockchain technology. The buyer’s accounts payable digitalization will be anchored by Linklogis technology, resulting in digital payment responsibilities. N-tier suppliers have three choices when they receive digital payment obligations: (1) They have the ability to hold till maturity, and anchor purchasers have the ability to pay. (2) They have the option of transferring all or a portion of it to the next Tier provider. (3) They can get a discount at the bank. This technology has the benefit of being able to be dismantled into small pieces. The N-level provider retains the high-quality credit of anchoring the customer even after receiving the requirement to split the digital payment. Small and medium-sized businesses that participate in digital payment obligations can benefit from cheaper loan rates and quicker access to capital. The beauty of this proposal is that it excludes personal finance for second- and third-tier SMEs in the supply chain. They can receive funding fast after joining the deep-level finance mechanism. Collaboration with industry partners is helpful for cross-border in-depth funding.

Blockchain is no longer P2P or decentralized, having developed from Bitcoin. This technology has the ability to revolutionize how businesses are run and serviced. The speaker undoubtedly offered in-depth insights into how blockchain might assist financial institutions and other partners in lowering costs, lowering risks, automating operations, and promoting the future development of SMEs.

### What Advantages Can Blockchain Bring to the Financing of Small and Medium Enterprise’s in the Kingdom?

Blockchain is expected to offer several distinct benefits to small and medium-sized businesses, including increased trust, speed, and security, as well as a reduction in the risk of identity fraud and hacker assaults, saving time and money.

This might help them handle cash flow difficulties, paperwork challenges, and worldwide issues (owing to blockchain platforms’ globalization), preventing companies from going bankrupt.

#### Availability of Fund

To begin with, the chance of running out of money will be substantially decreased. Because there is no uncertainty about when the money will be released, the firm can offer services on schedule, knowing that the funds will be there when they are needed. It may become easier to pay for goods and pay wages to overseas employees from distant buyers and can be done at a fraction of current costs. Therefore, it can help bring products and transaction services to the market quickly and cheaply.

#### Transactions Are Safer and More Assured

Enterprises will benefit from blockchain’s value-added features such as security and transparency. Blockchain technology combined with secure communication technology can assure the safety and security of transactions for small and medium-sized businesses with global ambitions.

Information asymmetries, mortgage requirements, a lack of competent credit reporting agencies, Internet data security, and cybercrime will all be addressed by blockchain technology. As a result, blockchain technology can provide secure, automated, and efficient data transfers, which may be used to trade private information, track the source of food, and monitor items in transit.

#### High Cost-Effective Process

In order to improve process efficiency, blockchain applications will streamline corporate operations and offer significant cost and process complexity reduction possibilities. The major benefit of small companies hosting services on the blockchain is that indirect expenses are significantly reduced. Using blockchain reduces the amount of money and time entrepreneurs spend on management activities. This may reduce security expenses, Know Your Customer (KYC) agreements, data storage costs, and other overheads.

The cost savings may be passed on to customers, making pricing more competitive, in addition to considerably lowering the investment that founders must make in these support operations. This creates a fairer playing field for small and medium-sized businesses all around the world.

### How Blockchain Solves the Credit Gap: Credit Availability for Small and Medium Enterprises

New platforms and business models have arisen as a result of the advent of massive data pools, machine learning, and venture capital interest in financial technology, providing credit solutions for small and medium-sized businesses. These financial technologies are growing rapidly, but the amount of credit and the scope of the market are still limited compared to the amount of credit needed to influence economic activity in a meaningful way. However, new blockchain solutions will greatly increase the chances of obtaining credit. In this article, we reviewed current SME loan solutions and explored, how blockchain and distributed ledger technology (DLT) may help people get more money and close credit gaps.

#### Solutions of FinTech for Small and Medium Enterprise Credit

In the past 10 years, to meet the financing demands of SMEs, FinTech start-ups have developed a range of business models. These platforms may be roughly split into three categories:

◾ Platforms for peer-to-peer lending, such as Lending Club, allow capital suppliers to select particular credit opportunities that the firm seeks.
◾ Companies can get term loans and other kinds of funding through SME balance sheet loans, such as Kabbage.
◾ Companies that offer invoice financing, such as Aprilia and Lydia, can sell or finance invoices.

These business models and products have become viable because to advances in machine learning, data pools derived from company records, online accounting systems, and information gleaned from public sources and social media. Higher interest rates are charged by service providers, but because the periods are often short, the borrower is unaware that the yearly interest rate might be as high as 25% or higher.

Furthermore, even the most forward-thinking FinTech start-ups are unprepared to lend to higher-risk borrowers, whether owing to a lack of data or a proprietary risk assessment based on comprehensive information, which leads to increasing hazards. As a result, financial product innovation is required to enable more SMEs to get financing.

#### Blockchain Solutions for Credit Needs

Blockchain and distributed ledger technologies bring trust and the capacity to communicate data, information, and proof of many things safely and reliably. This means that even if entities don’t trust one other, they may trust a common ledger that is immutable and includes unchangeable data.

Blockchain technology is already being used in trade finance, and companies like **we.trade** are collaborating to build a comprehensive trading ecosystem that includes documentation, communications, and settlement. Such platforms are tailored to specific use cases and enable existing banks and service providers to participate, at least in part, in the industry’s future. Adoption appears to be gradual, given the high cost of services and the complexity of trade financing.

The potential for innovation through shared trustworthy blockchain ledgers in supply chain finance are apparent and vast. Consider the possibility that any business can reach a successful agreement with its suppliers on the status of shipments, payments, and overdue services. The level of efficiency that can be achieved is nearly unbelievable. Foxconn launched an early test study for the first three layers of its supply chain as early as 2017. The program will be moved to an Ethereum-based platform with smart contract execution capabilities, according to the firm (referred to as “chained”).

Foxconn enables third-party finance providers to offer operations for their suppliers Fund funding by deploying a trusted universal ledger to validate the company’s outstanding orders for its suppliers (in an authorized, i.e., secure, and controlled access environment). Furthermore, Foxconn suppliers will not have to contact factoring service providers on their own, which will be more expensive than Foxconn’s platform advertising.

Why is supply chain financing a good place to start with blockchain? Because all parties may exchange a wealth of data and knowledge. Take, for example, Foxconn. It began in a restricted fashion, with the need that any participating provider be a recognized Foxconn supplier on its platform (imagine a “intranet” in the 1990s — or the 1980s). Such apps will be created in the future in huge open access settings (think of them as the “Internet”), where anybody with access may readily access encrypted data. We’ll keep track of supplier dependability, physical delivery evidence, service delivery proof, quality standards proof, and other essential company operations. Loan choices will be made in a range that is now inconceivable based on these validated facts.

## Current Challenges and Solutions for Saudi Small and Medium Enterprise Financing

### Credits (Loans) From Banks

Obtaining loans from financial institutions may be difficult for SMEs, particularly for those who are just starting out. Due to shortage of funds, the lack of opportunities has resulted in limited survival opportunities for many SME’s have closed in the first 3 years of operation.

Since the banking crisis in 2008, banks have essentially avoided risks. As a result, they have a limited tolerance for lending to SMEs. According to a survey by the International Finance Corporation (IFC), 40% of formal microfinance as well as small and medium enterprises in emerging markets have an unmet financing demand of 5.2 trillion United States dollars (USD) each year.

### Funding

Obtaining trade credit is another issue that SMEs with worldwide operations confront. Trade financing, like many other types of credit, is critical to the success of SMEs, but it is not always simple to get. Obtaining traditional trade finance instruments, such as letters of credit, forfaiting (sales arrears at a discounted price in return for cash), or trade credit insurance in import and export firms, can be difficult for SMEs.

### Cash-Flow Problem

Small firms suffer greatly as a result of their inability to raise financing, which stifles expansion and produces cash flow problems. Companies require cash flow to purchase supplies, begin manufacturing processes, pay employees’ wages, and cover other operating costs. Late payment might be the difference between success and failure for small businesses.

### Partial Financing (Unconventional)

To get capital and ease cash flow difficulties, SMEs nowadays frequently turn to alternative financing options. Alternative financing mechanisms, such as peer-to-peer (P2P) or crowdfunding, have developed in recent years, with the latter focused mostly on technological start-ups, although the situation is mixed.

### For Small and Medium Enterprise Finance, Blockchain Technology Is a Viable Answer

Blockchain and distributed ledger technologies bring trust and the capacity to safely and reliably communicate data, information, and proof of many things. This means that entities who don’t necessarily trust each other can rely on a shared ledger that is immutable and includes unchangeable data.

Although most people identify blockchain with huge corporations, the technology also provides new options for SME financing in a variety of industries to address current issues and build new business models.

#### Finance (Trade)

Blockchain-based solutions are already being used in trade finance, and industry participants are working together to create a comprehensive trading environment that includes documentation, communications, and settlement. Trade finance solutions are becoming more efficient as transparency and consensus procedures supplant numerous verifications and inspections.

Blockchain technology, according to a joint research by the World Economic Forum and consulting company Bain & Company, can help bridge the global trade finance divide and promote commerce that would otherwise be impossible.

#### Supply Chain Finance

The use of blockchain technology might also aid in the acquisition of supply chain financing (SCF). Nowadays, a bigger market sector is developing open account solutions, but because assessing the depth of the supply chain is challenging, funding is generally limited to a few tiers. Because blockchain is considerably more versatile than conventional digital systems when it comes to processing data, it offers up new opportunities for other chain players to get SCF.

Suppliers and purchasers may both view transaction information in real time on the blockchain. All stakeholders time stamp and verify each stage of the supply chain process, ensuring that the data is correct and unchangeable. This enhanced visibility might also imply that firms will have additional options for invoice finance. This openness may hasten transaction processing, increase supplier cash flow, and raise interest rates for invoice finance companies.

The potential for innovation through shared trusted blockchain ledgers; are both clear and enormous in SCF. Consider how quickly and simply a firm might reach an agreement with its suppliers about the status of shipments, payments, and overdue services. The increase in efficiency is enormous.

#### Agreements (Smart Contracts)

One of the most appealing aspects of blockchain is its ability to enable smart contract for small businesses. It not only outlines the agreement’s terms and penalties in the same manner that traditional contracts do, but it also enforces and enforces those pre-agreed terms and conditions automatically (There is no need for an intermediary agency). Many labor-intensive and costly corporate procedures may be quickly and inexpensively replaced in this way.

The most significant advantage of smart contracts is the creation of a single digital record of customs clearance. A smart contract can also be used as collateral to back a loan by representing an invoice or other comparable financial document. Smart contracts will aid in the reduction of credit risk, the reduction of costs, and the removal of trade obstacles.

#### Financing: Type of Collateral

In terms of securing funding for small businesses, blockchain technology has the potential to revolutionize the way we do things. By automating what was formerly a laborious process, enhancing transparency, and encouraging confidence between lenders and borrowers, blockchain can help revive P2P lending practices.

The blockchain’s disintermediation feature makes it easier and faster for SMEs to obtain money through shares. Removing these roadblocks reduces the need for complicated paperwork, and the automated nature of the process may allow commissions, excessive brokerage fees associated with stock sales, and other administrative costs to be avoided.

### Build a Future Technology Designed for Small and Medium Enterprise’s Loans

Small and medium enterprise loans in the future will need to integrate financial technology innovation from the previous decade with blockchain technology that is just getting started. Capital providers may now make lending choices using more data, stronger algorithms, and a trading environment that supports APIs thanks to financial technology innovation. More cash is being invested in SMEs thanks to the rise of crowdfunding and peer-to-peer financing.

I foresee substantial changes in SME loans and the capacity of SMEs to acquire credit as a result of combining these advancements with the trust and verification engine provided by blockchain-enabled apps. The first creative solution was implemented in a private, permissioned environment (similar to the “intranet” in the 1990s), serving only a small number of users and severely restricting credit. Many basic technological concerns, such as zero-knowledge proof and metadata tracking, must be overcome before we shift to a public encryption environment. Innovative businesses like **param.network** are already integrating the benefits of open source technology with an on-demand licensing layer to provide an accessible yet controlled environment, which is exactly what corporate clients need.

Large firms looking to stabilize their supply chains and provide third-party working capital finance for their suppliers will drive supply chain financing innovation. Corporate groupings and coalitions trying to preserve some control over complicated but valuable business lines will drive trade finance innovation.

Standardized financial instruments ***(for example, based on ACTUSFRF)*** issued locally on the blockchain will allow all parties to resort to the same computations and verified and audited cash flows. Such standards will enable for near-real-time reporting and the securitization of loan portfolios that were previously thought to be impossible to securitize.

### There Is a Need for a Regulatory System

Block-chain SMEs confront unclear legislation, which restricts their options and jeopardizes their growth. The legality of smart contracts and the worldwide regulatory framework necessary to enable meaningful cross-border peer-to-peer lending will be the main problem in the future; what is lawful in one nation may not be legal in another.

A “good” regulatory framework should be more transparent, encourage adoption, and prevent fraud, such as that using user anonymity in transactions. At the same time, blockchain and smart contracts’ strength and potential are becoming more widely acknowledged in the corporate and political worlds. Although authorities may take some time to catch up, widespread use will lead to more sensible regulation.

## Look Forward To: A Futuristic Approach

Small and medium enterprise finance in the future will need a combination of financial technology, financing strategies, and developing blockchain technology innovation. Blockchain-based applications track payments, contract fulfillments, and a variety of other business activities, allowing capital providers to make loan decisions based on more data, improved algorithms, and application programming interfaces (APIs), which define how different software and devices interact in the trading environment.

With these benefits in mind, it’s easy to see why a growing number of SME businesses are ready to invest more in blockchain technology. The SME community may wish to see a total shift in the way they do business now with the aid of associated services such as blockchain and smart contracts. International transactions will be more beneficial to SMEs, and they may be able to compete in ways that are currently unthinkable.

Blockchain, on the other hand, is still in its infancy. The use of blockchain by SMEs on a big scale has yet to begin, and mainstream acceptance will take time. The most difficult hurdle to overcome is allowing more firms to develop on the blockchain and encouraging customers to utilize these solutions. This need faith.

Trust takes time to develop, and some businesses must begin the process of trusting in order to make promises a reality. Demonstrate to the rest of the world that blockchain can be used for anything relevant to business. Given that this technology will increase commerce by more than $1 trillion in the next 10 years, according to the World Economic Forum, major blockchain businesses may be compelled to provide solutions that are more beneficial to SMEs.

Standardized financial tools developed in a digital environment enhanced by the blockchain will enable all parties to refer to the same calculations and verified and audited cash flows. Such a standard would produce near real-time reports and allow the securitization of loan portfolios, which was previously considered impossible.

The use of blockchain technology, which is driven by the asset digitization process, can not only totally transform the way we think about risk, but also bring novel solutions for pricing, hedging, and risk management in the financial market and across the real economy. Its restructuring will gradually improve lenders’ capacity to give loans to borrowers with bad credit and re-establish trust in doing so.

## Conclusion

Blockchain as a technology has gained a lot of attention in the past few years. Using this technology to finance Saudi SMEs may solve many of the difficulties faced by SMEs. Agriculture, insurance, education, health and pharmaceuticals, technology and utilities are just a few industries where this technology may benefit SMEs. The institutional factors that use blockchain technology to fund Saudi SMEs provide benefits such as cost-effectiveness, transparency, and the kingdom’s growth potential. On the other hand, small businesses that want to use blockchain technology should be cautious and weigh the pros and cons carefully.

Even if a small group of small and medium-sized enterprises successfully incorporate blockchain technology into their business strategy, without a solid and larger network, the benefits of this technology are not enough for them. Therefore, more SMEs should be funded through this network to optimize their interests in the Kingdom of Saudi Arabia. There are many institutional factors for funding Saudi SMEs. Global and national trade unions or organizations should promote and recommend Saudi Arabia’s SMEs to adopt blockchain technology. They should provide incentives and knowledge management plans to encourage them to use blockchain technology to accept and target these determinants in the company’s daily work.

In addition, because this technology is new, global and national policy makers have a lot of uncertainty about how to manage it. Due to possible money laundering investigations, the lack of universally accepted legislation will inevitably bring uncertainty and hesitation to companies seeking to benefit from blockchain technology. In order to reduce the ambiguity surrounding the blockchain, the International Trade Organization should propose a global regulatory framework as soon as possible so that more companies can use it safely.

All in all, this research is the first step to incorporate blockchain technology into institutional variables that influence the use of blockchain technology to fund future projects for Saudi SMEs. Based on the ideas and framework provided in the article, various potential research paths may be generated to examine the applicability of the institutional determinants of financing Saudi SMEs using blockchain technology.

## Implications

### Implications for Theory

This is rare study on the emerging field in the literature on kingdom which is a call for attention of the researchers of the region and similar domain to explore further perspectives of the blockchain and how to pave its way for the economic expansion.

### Implications for Practice and Policy

Government agencies to formulate policies and programs to support and promote SME’s in the better interest of the social and economic development. However, financial institutions and other relevant organizations need to undertake responsibility and come forward for the cause.

## Data Availability Statement

The original contributions presented in the study are included in the article/supplementary material, further inquiries can be directed to the corresponding author.

## Author Contributions

NA conceptualized the manuscript and contributed to original writing. MT contributed to original writing of the manuscript, editing, and formatting. Both authors contributed to the article and approved the submitted version.

## Conflict of Interest

The authors declare that the research was conducted in the absence of any commercial or financial relationships that could be construed as a potential conflict of interest.

## Publisher’s Note

All claims expressed in this article are solely those of the authors and do not necessarily represent those of their affiliated organizations, or those of the publisher, the editors and the reviewers. Any product that may be evaluated in this article, or claim that may be made by its manufacturer, is not guaranteed or endorsed by the publisher.
