# Corrigendum: Role of leadership in adoption of blockchain technology in small and medium enterprises in Saudi Arabia

**DOI:** 10.3389/fpsyg.2022.1052380

**Published:** 2022-11-28

**Authors:** Nasser Alshareef, Muhammad Nawaz Tunio

**Affiliations:** ^1^College of Business Administration (CBA), Majmaah University, Al Majma'ah, Saudi Arabia; ^2^Department of Management Science, Mohammad Ali Jinnah University, Karachi, Pakistan

**Keywords:** SME's, financing, blockchain, Saudi Arabia, distributed ledger technology (DLT)

In the published article, there was an error in affiliation 2. Instead of “^2^ Department of Business Administration, Greenwich University, Karachi, Pakistan,” it should be, “^2^ Department of Management Science, Mohammad Ali Jinnah University, Karachi, Pakistan.”

In the published article, there was an error in the Funding statement. The correct Funding statement appears below.

## Funding

The authors would like to thank the Deanship of Scientific Research at Majmaah University for supporting the work under Project Number. R-2022-213.

The authors apologize for these errors and state that this does not change the scientific conclusions of the article in any way. The original article has been updated.

## Publisher's note

All claims expressed in this article are solely those of the authors and do not necessarily represent those of their affiliated organizations, or those of the publisher, the editors and the reviewers. Any product that may be evaluated in this article, or claim that may be made by its manufacturer, is not guaranteed or endorsed by the publisher.

